# Multi-level, cross-sectional study of workplace social capital and smoking among Japanese employees

**DOI:** 10.1186/1471-2458-10-489

**Published:** 2010-08-17

**Authors:** Etsuji Suzuki, Takeo Fujiwara, Soshi Takao, S V Subramanian, Eiji Yamamoto, Ichiro Kawachi

**Affiliations:** 1 Department of Epidemiology, Okayama University Graduate School of Medicine, Dentistry and Pharmaceutical Sciences, 2-5-1 Shikata-cho, Kita-ku, Okayama 700-8558, Japan; 2 Section of Behavioral Science, Department of Health Promotion, National Institute of Public Health, 2-3-6 Minami, Wako-shi, Saitama 351-0197, Japan; 3 Department of Society, Human Development, and Health, Harvard School of Public Health, 677 Huntington Avenue, Boston, MA 02115, USA; 4 Department of Information Science, Faculty of Informatics, Okayama University of Science, 1-1 Ridai-cho, Kita-ku, Okayama 700-0005, Japan

## Abstract

**Background:**

Social capital is hypothesized to be relevant to health promotion, and the association between community social capital and cigarette smoking has been examined. Individual-level social capital has been found to be associated with smoking cessation, but evidence remains sparse on the contextual effect of social capital and smoking. Further, evidence remains sparse on the association between smoking and social capital in the workplace, where people are spending an increasing portion of their daily lives. We examined the association between workplace social capital and smoking status among Japanese private sector employees.

**Methods:**

We employed a two-stage stratified random sampling procedure. Of the total of 1,800 subjects in 60 companies, 1,171 (men/women; 834/337) employees (65.1%) were identified from 46 companies in Okayama in 2007. Workplace social capital was assessed in two dimensions; trust and reciprocity. Company-level social capital was based on inquiring about employee perceptions of trust and reciprocity among co-workers, and then aggregating their responses in order to calculate the proportion of workers reporting mistrust and lack of reciprocity. Multilevel logistic regression analysis was conducted using Markov Chain Monte Carlo methods to explore whether individual- and company-level social capital was associated with smoking. Odds ratios (ORs) and 95% credible intervals (CIs) for current smoking were obtained.

**Results:**

Overall, 33.3% of the subjects smoked currently. There was no relationship between individual-level mistrust of others and smoking status. By contrast, one-standard deviation change in company-level mistrust was associated with higher odds of smoking (OR: 1.25, 95% CI: 1.06-1.46) even after controlling for individual-level mistrust, sex, age, occupation, educational attainment, alcohol use, physical activity, body mass index, and chronic diseases. No clear associations were found between lack of reciprocity and smoking both at the individual- and company-level.

**Conclusions:**

Company-level mistrust is associated with higher likelihood of smoking among Japanese employees, while individual perceptions of mistrust were not associated. The link between lack of reciprocity and smoking was not supported either at the individual- or company-level. Further studies are warranted to examine the possible link between company-level trust and smoking cessation in the Japanese workplace.

## Background

Social capital is defined as the resources - such as trusting relations and norms of reciprocity - available within social networks and social structures [1-3]. Within public health research, the literature has evolved around two distinct conceptions of social capital, one that emphasizes processes and outcomes associated with social cohesion (e.g., trust, solidarity, norms), and the other that emphasizes resources accessed through social networks [4]. The social cohesion definition tends to emphasize social capital as a group attribute and to analyze it as a contextual influence on individual health [1]. By contrast, the network theory of social capital defines the concept in terms of resources (e.g., social support), and it employs methods of sociometric analysis, i.e., either ego-centered network mapping or whole network analysis [5]. These two approaches are not mutually exclusive [4,6].

Social capital is hypothesized to be relevant to health promotion because the same resources can be mobilized to achieve better health outcomes [1-3]. For example, a more cohesive neighborhood (or workplace) is better equipped to undertake collective action to restrict smoking in public places [7]. Cohesive social structures are also more effective in enforcing norms (e.g., making sure that members do not smoke in restricted places) and enforcing sanctions. Finally, the diffusion of innovations (for example, information about smoking cessation aids) is likely to occur faster within cohesive social structures. In support of these assertions, a growing body of empirical research has suggested the beneficial effects of social capital on a range of health outcomes [2], including mortality [8,9], physical health (e.g., cardiovascular disease) [10,11], and mental health [12].

Individual-level social capital has been found to be inversely associated with daily smoking [13,14], and aspects of social capital, such as social participation [15-17], have been also associated with smoking cessation. With regard to neighborhood-level social capital and smoking, however, the evidence is limited, and has been more mixed. A multilevel study in England [18] indicated that community social capital was associated with lower levels of smoking. A contextual econometric analysis in the US [19] indicated that the proportion of community social capital attributable to religious groups (i.e., if the strong nonsmoking norms exist in communities) was inversely and strongly related to the number of cigarettes that smokers consumed, while it was not attributable to the prevalence of smoking. In a multilevel study in Sweden [20], however, no remaining neighborhood variance was found in daily smoking after adjustments for individual compositional factors. Indeed, social capital can be a "double-edged sword" depending upon exactly what the local norms are promoting (e.g., smoking in order to promote social bonding). On the other hand, if the goal is to introduce a health-promoting innovation - such as a smoking cessation intervention - theory would predict that this would be facilitated by stronger trust and reciprocity within the group [7,21].

As employee smoking is not only harmful to health but also very costly (e.g., absenteeism, worker's compensation payment, property damage, etc.) [22], it is worth knowing whether workplace social capital can promote smoking cessation among workers. However, evidence on the link between workplace social capital and smoking status remains sparse. Kouvonen *et al*. [23] examined the association between workplace social capital and smoking cessation among Finnish public sector employees and reported that workers reporting a high level of social capital were more likely to quit smoking during a mean follow-up of 3.6 years, particularly among high socioeconomic workers. However in that study, although individual perceptions of social capital were associated with smoking cessation, the work-unit level of social capital (aggregated from individual responses) was not associated with smoking cessation. However, that study is based on a female-dominated (> 70%) cohort of public sector employees, some of whom were employed in hospitals. Thus further studies are warranted to examine the relationships based on employees in different workplaces (e.g., private sectors) and in different cultural settings (e.g., Japan). Indeed, the meaning of workplace social capital may be influenced by cultural background (e.g., values of workplaces) [24-26].

Accordingly, in the present study we sought to examine the association between workplace social capital (both at individual- and company-levels) and smoking status among Japanese private sector employees.

## Methods

The study was based on a representative survey of employees in Okayama prefecture, which is located west of Osaka, in 2007. The local prefectural government sought to conduct a survey to examine how the companies have established health management systems in the workplaces. By piggy-backing on to this survey, we managed to add some additional questions related to social capital in the workplace to test the hypotheses examined in the present study. A two-stage stratified random sampling procedure was employed in which we targeted 60 companies headquartered in Okayama. Companies were stratified into three categories according to the number of their employees; 50 to 99, 100 to 299, and 300 or more employees. The numbers of companies in each category were 92, 104, and 57, respectively. We randomly selected 20 companies in each stratum. In the second stage, we randomly sampled 30 employees from each company. To standardize the sampling procedure, we instructed the Human Resources department of each company on how to randomly sample employees from their payroll. Part-time workers were included, while board members were excluded to limit our investigation to the general employees. Questionnaires were distributed to each selected participant, and returned in anonymous, sealed envelopes.

In the present study, workplace social capital was conceptualized and measured in two dimensions: trust and reciprocity among co-workers. Workplace trust was assessed by asking the following question, which was derived from the US General Social Survey item and was slightly modified by specifying the reference unit (the company) [27]: "Generally speaking, would you say that most people in your company can be trusted or that you can't be too careful in dealing with people?" Responses were obtained according to four predetermined categories (1 = Most people can be trusted, 2 = Can't be too careful, 3 = Other, 4 = Don't know). Similarly, collective perceptions of reciprocity in the workplace were tapped by the following question: "Would you say that most of the time people in your company try to be helpful, or that they are mostly just looking out for themselves?" Responses were obtained according to four predetermined categories (1 = Try to be helpful, 2 = Just look out for themselves, 3 = Other, 4 = Don't know). Employees reporting mistrust and lack of reciprocity were defined as belonging to low social capital workplaces ("Exposed"). The "Other" and "Don't know" categories were modeled as dummy variables. Company-level social capital was based on aggregating employee responses and calculating the proportion of workers reporting mistrust and lack of reciprocity.

Smoking status was assessed by the following question: "How many cigarettes do you smoke a day on average now?" Responses were obtained according to five predetermined categories (never, former, 1-10 cigarettes/day, 11-20 cigarettes/day, and ≥ 21 cigarettes/day). From this item, we created a dichotomous outcome measure (never/former vs. current). Self-reported smoking status has been validated in several studies [28,29].

We selected the following variables as relevant confounders for statistical control: sex, age (10-year categories), occupational status (non-manual vs. manual), education (less than senior high school education vs. more advanced educational attainment), frequency of alcohol consumption, and leisure-time physical activity (any vs. none). Regarding age, the two oldest categories were collapsed into one category (60 or older) due to sparse data. Alcohol consumption was categorized into two groups as follows: low (none, rarely, 1-3 days/month) vs. high (1-2 days/week, 3-4 days/week, 5-6 days/week, everyday). In addition, height and weight were reported, from which body mass index (BMI) was calculated and dichotomized at 25 kg/m^2 ^according to the guideline of the Japan Society for the Study of Obesity. We also created indicator variables for whether the respondent had been told by their physician that they had one of the following chronic conditions during recent health checkups: high blood sugar, high blood pressure, and low high-density lipoprotein (HDL) and/or high triglyceride.

A separate survey was administered to the representatives of each company to inquire about the degree of implementation of smoking restriction policies in their company. The responses were obtained according to five predetermined categories (very good, good, neutral, poor, and very poor).

The study was reviewed and approved by the Office of Human Research at the Harvard School of Public Health.

### Statistical analyses

Our data had a multilevel structure comprised of employees (at level 1) nested within companies (at level 2). We fitted the data using multilevel logistic regression models, adjusting for both employee- and company-level variables as fixed effects and allowing for heterogeneity between companies [30]. Company-level random effect of the intercept was assumed to be normally distributed with a mean of zero.

After examining the company-level variance in smoking without including any explanatory variables (empty model), we examined the relationship between individual-level social capital and current smoking by adjusting for other individual-level covariates (model 1). Next, we included company-level social capital index instead of individual-level social capital into the model 1 (model 2). Then, we modeled individual- and company-level social capital variables simultaneously (model 3). Finally, we added cross-level interaction terms between company-level social capital and individual-level social capital (model 4); company-level social capital and sex (model 5); company-level social capital and age (model 6); company-level social capital and occupation (model 7); and company-level social capital and educational attainment (model 8). In models 3 to 8, individual-level mistrust and lack of reciprocity were centered around the company mean (i.e. the proportion of the employees reporting mistrust and lack of reciprocity, respectively) to make them orthogonal, thus overcoming the problem of collinearity between individual-level and company-level social capital indicators [30,31]. Further, by employing the group-mean centering procedure, we were able to disentangle the "pure" individual versus contextual effects of social capital on smoking [30,31]. As a supplementary analysis, we added a smoking restriction policy and a company size as company-level ordinal variables.

We applied Bayesian inference to estimate the parameters, using Markov Chain Monte Carlo (MCMC) methods using MLwiN 2.1 software. We used non-informative Gaussian priors for the fixed effects and non-informative uniform priors for the between-company variances. On the basis of the Raftery-Lewis diagnostic, we monitored the length of Markov chain required for the convergence, discarding the first 500 simulations as burn-in. Based on the mean as well as the 2.5th and 97.5th percentiles of the posterior distributions, odds ratios (ORs) and 95% credible intervals (CIs) for current smoking were obtained for each variable. For company-level social capital, we calculated ORs associated with one-standard deviation change in the corresponding variables. With regard to company-level variance, median odds ratios (MORs) were calculated [32,33]. If the MOR is 1, there is no variation between companies. If there is considerable between-company variation, the MOR will be large. The measure is directly comparable with fixed-effects ORs. We used the Deviance Information Criterion (DIC) to compare the goodness-of-fit of each model [34].

## Results

Of the total of 1,800 subjects, questionnaires were returned from 1,218 employees (67.6%) from 46 companies (16, 15, and 15 from each stratum). The mean response rate among these 46 companies was 83.1% (median; 90.0%, range from 36.7% to 100%). We excluded respondents with missing values on the social capital questions, smoking status, sex, or age, which resulted in 1,171 subjects (65.1%) available for the present study. The demographic characteristics and the proportion of smoking subjects are shown in table 1. No significant differences were observed in any of the variables in table 1 between the analyzed and the excluded subjects. Overall, 33.3% of the subjects smoked currently. No substantial differences were observed in the smoking prevalence according to the responses to trust and reciprocity.

**Table 1:** Demographic characteristics and smoking status of the study subjects

Characteristics	No.	%	Current smokers	
			
			No.	%
All	1171	100.0	390	33.3
Social capital (trust)				
Trust	537	45.9	172	32.0
Mistrust	199	17.0	69	34.7
Other	168	14.3	62	36.9
Don't know	267	22.8	87	32.6
Social capital (reciprocity)				
Reciprocity	292	24.9	101	34.6
Lack of reciprocity	328	28.0	113	34.5
Other	185	15.8	66	35.7
Don't know	366	31.3	110	30.1
Sex				
Men	834	71.2	359	43.0
Women	337	28.8	31	9.2
Age				
Younger than 20	15	1.3	3	20.0
20 to 29	207	17.7	72	34.8
30 to 39	348	29.7	112	32.2
40 to 49	317	27.1	112	35.3
50 to 59	250	21.3	82	32.8
60 to 69	33	2.8	9	27.3
70 or older	1	0.1	0	0.0
Occupational status				
Non-manual	1014	86.6	330	32.5
Manual	142	12.1	52	36.6
Missing	15	1.3		
Education^a^				
Higher	668	57.0	207	31.0
Basic	502	42.9	182	36.3
Missing	1	0.1		
Alcohol consumption^b^				
Lower	593	50.6	143	24.1
Higher	575	49.1	245	42.6
Missing	3	0.3		
Physical activity				
Yes	395	33.7	140	35.4
No	758	64.7	243	32.1
Missing	18	1.5		
Body mass index (kg/m^2^)				
Less than 25	886	75.7	301	34.0
25 or larger	233	19.9	83	35.6
Missing	52	4.4		
High blood sugar				
No	1074	91.7	354	33.0
Yes	81	6.9	31	38.3
Missing	16	1.4		
High blood pressure				
No	997	85.1	336	33.7
Yes	158	13.5	49	31.0
Missing	16	1.4		
Low high-density lipoprotein (HDL) and/or high triglyceride				
No	863	73.7	289	33.5
Yes	292	24.9	96	32.9
Missing	16	1.4		

^a ^Basic education is defined as less than senior high school education, and higher education is defined as more advanced educational attainment.

^b ^Lower alcohol consumption category includes none, rarely, and 1-3 days a month. Higher category includes 1-2 days a week, 3-4 days a week, 5-6 days a week, and every day.

The mean proportions of reporting mistrust and lack of reciprocity in each company were 18% and 28%, respectively. The proportion of smokers in each company ranges from 10% to 63%. When we examined the associations between the proportion of workers reporting mistrust and lack of reciprocity and the smoking prevalence of the 46 companies, a moderately positive relationship was observed with mistrust (r = 0.27, p = 0.07), but no clear pattern was observed with lack of reciprocity (r = -0.03, p = 0.82).

Table 2 shows the ORs and 95% CIs for smoking associated with workplace mistrust. In empty model, the MOR of empty model was 1.54 (95% CI: 1.26-1.87), indicating that in the median case the residual heterogeneity between companies increased by 1.54 times the individual odds of smoking when randomly selecting two workers in different companies. When we examined the relationship between individual mistrust and smoking (model 1), no association was found (OR: 1.03, 95% CI: 0.69-1.53). By contrast, when we examined the relationship between company-level mistrust and smoking (model 2), we found that company-level mistrust was associated with higher odds of smoking (OR: 1.23, 95% CI: 1.06-1.44). These results did not change substantially when we examined individual- and company-level mistrust simultaneously (model 3). When we further examined cross-level interactions, we could not find any clear associations (data not shown).

**Table 2:** The ORs for current smoking associated with individual-level and company-level mistrust at work^a^

	Empty model	Model 1	Model 2	Model 3
				
	OR (95% CI)	OR (95% CI)	OR (95% CI)	OR (95% CI)
**Fixed effects**				
*Individual-level variables*				
Individual mistrust (vs. trust)^b^		1.03 (0.69-1.53)		0.90 (0.60-1.35)
Women (vs. men)		0.14 (0.09-0.22)	0.14 (0.09-0.22)	0.13 (0.08-0.21)
Age (in 10 years unit)		0.85 (0.74-0.98)	0.86 (0.76-0.99)	0.87 (0.76-1.00)
Manual (vs. non-manual) work		0.82 (0.53-1.29)	0.87 (0.57-1.34)	0.85 (0.55-1.32)
Basic (vs. higher) educational achievement		1.52 (1.11-2.06)	1.47 (1.08-1.97)	1.44 (1.06-1.95)
High (vs. low) alcohol use		1.67 (1.24-2.26)	1.69 (1.26-2.29)	1.68 (1.25-2.25)
No (vs. yes) physical activity		0.98 (0.73-1.31)	0.97 (0.73-1.30)	0.99 (0.74-1.33)
25 or larger (vs. < 25) BMI (kg/m^2^)		0.90 (0.63-1.27)	0.89 (0.63-1.26)	0.90 (0.64-1.28)
High blood sugar		1.16 (0.68-1.95)	1.11 (0.65-1.87)	1.15 (0.67-1.92)
High blood pressure		0.73 (0.48-1.12)	0.72 (0.47-1.09)	0.71 (0.46-1.08)
Low HDL and/or high triglyceride		0.75 (0.54-1.04)	0.74 (0.53-1.03)	0.74 (0.53-1.03)
*Company-level variable*				
Company-level mistrust^c^			1.23 (1.06-1.44)	1.25 (1.06-1.46)
**Random effects**				
Company-level variance (SD)	0.204 (0.094)	0.073 (0.068)	0.042 (0.052)	0.041 (0.047)
Median odds ratio	1.54 (1.26-1.87)	1.29 (1.03-1.60)	1.22 (1.03-1.51)	1.21 (1.03-1.48)
Deviance Information Criterion	1470.45	1254.05	1246.95	1248.56

BMI, body mass index; CI, credible interval; HDL, high density lipoprotein; OR, odds ratio; SD, standard deviation.

^a ^All models except for empty model are adjusted for sex, age, occupational status, education, alcohol consumption, physical activity, body mass index, and co-morbid conditions (high blood sugar, high blood pressure, and low HDL and/or high triglyceride).

^b ^Individual-level mistrust was group-mean centered in model 3.

^c ^Company-level mistrust was defined as the proportion of the employees reporting mistrust within the company. The odds ratios associated with a one-standard deviation change in the company-level mistrust (9.8%) are shown.

In table 3, individual lack of reciprocity was not associated with smoking (OR: 1.17, 95% CI: 0.80-1.71) (model 1). When we examined the relationship between company-level lack of reciprocity and smoking, we found no clear association (model 2). These results did not change substantially when we examined individual- and company-level lack of reciprocity simultaneously (model 3). In further analyses, a marginally significant cross-level interaction was observed between company-level lack of reciprocity and age (OR: 0.36, 95% CI: 0.13-1.01), indicating that age is associated with higher odds of smoking in companies with high reciprocity (OR: 1.14) and lower odds of smoking in companies with low reciprocity (OR: 0.41; which is computed by multiplying 1.14 by 0.36).

**Table 3:** The ORs for current smoking associated with individual-level and company-level lack of reciprocity at work^a^

	Empty model	Model 1	Model 2	Model 3
				
	OR (95% CI)	OR (95% CI)	OR (95% CI)	OR (95% CI)
**Fixed effects**				
*Individual-level variables*				
Individual lack of reciprocity (vs. reciprocity)^b^		1.17 (0.80-1.71)		1.15 (0.79-1.69)
Women (vs. men)		0.14 (0.09-0.22)	0.14 (0.09-0.22)	0.14 (0.09-0.22)
Age (in 10 years unit)		0.84 (0.74-0.97)	0.85 (0.75-0.97)	0.84 (0.74-0.97)
Manual (vs. non-manual) work		0.85 (0.54-1.33)	0.85 (0.55-1.32)	0.85 (0.55-1.34)
Basic (vs. higher) educational achievement		1.53 (1.13-2.08)	1.52 (1.12-2.06)	1.52 (1.12-2.07)
High (vs. low) alcohol use		1.68 (1.25-2.28)	1.67 (1.25-2.25)	1.68 (1.25-2.26)
No (vs. yes) physical activity		0.98 (0.73-1.30)	0.97 (0.73-1.29)	0.98 (0.73-1.30)
25 or larger (vs. < 25) BMI (kg/m^2^)		0.90 (0.63-1.28)	0.90 (0.64-1.28)	0.90 (0.64-1.28)
High blood sugar		1.15 (0.68-1.94)	1.13 (0.67-1.89)	1.15 (0.68-1.94)
High blood pressure		0.73 (0.48-1.11)	0.73 (0.48-1.11)	0.73 (0.48-1.10)
Low HDL and/or high triglyceride		0.75 (0.54-1.04)	0.75 (0.54-1.04)	0.75 (0.54-1.04)
*Company-level variable*				
Company-level lack of reciprocity^c^			1.05 (0.89-1.23)	1.06 (0.89-1.24)
**Random effects**				
Company-level variance (SD)	0.204 (0.094)	0.076 (0.071)	0.078 (0.069)	0.068 (0.070)
Median odds ratio	1.54 (1.26-1.87)	1.30 (1.03-1.61)	1.31 (1.04-1.61)	1.28 (1.03-1.61)
Deviance Information Criterion	1470.45	1254.23	1252.28	1255.63

BMI, body mass index; CI, credible interval; HDL, high density lipoprotein; OR, odds ratio; SD, standard deviation.

^a ^All models except for empty model are adjusted for sex, age, occupational status, education, alcohol consumption, physical activity, body mass index, and co-morbid conditions (high blood sugar, high blood pressure, and low HDL and/or high triglyceride).

^b ^Individual-level lack of reciprocity was group-mean centered in model 3.

^c ^Company-level lack of reciprocity was defined as the proportion of the employees reporting lack of reciprocity within the company. The odds ratios associated with a one-standard deviation change in the company-level lack of reciprocity (11.2%) are shown.

When we further added a company-level variable of smoking restriction policy and a company size, no substantial changes were observed (data not shown). Overall, DIC values of the model 2 (both in tables 2 and 3) were smaller, suggesting better performance of this model.

## Discussion

In the present study, we examined the association between workplace social capital and smoking status among private sector employees. Our findings suggest that neither the mistrust of co-workers (as an individual perception) nor lack of reciprocity in the workplace is associated with workers' smoking status. By contrast, we found a contextual association between company-level mistrust and smoking status, even controlling for individual-level mistrust, sex, age, occupation, educational attainment, alcohol use, physical activity, body mass index, and chronic diseases. The same contextual association was not found between company-level lack of reciprocity and smoking.

In the only previously published study on this topic [23], higher individual-level social capital was shown to be associated with higher odds of smoking cessation, while work-unit social capital was not. In other words, the findings in the Finnish public sector employee study were opposite to the pattern we found. These studies examined different health behaviors (i.e., current smoking versus smoking cessation), which makes it difficult to compare their findings. Indeed, since this is only the second study on this topic, the discrepancy could be a chance finding. On the other hand, it is possible that the difference between the results of the Finnish study and our Japanese study may reflect broader cultural differences between western individualism versus Asian conformism [35]. Thus, given that smoking has been the social norm Asian men may be more likely to smoke if they have a high level of trust and reciprocity, whereas smoking cessation in Finland may be related to individual decisions to quit. Further, in the Finnish study, the finding that individual perceptions of social capital predicted smoking cessation (while aggregated perceptions did not) suggests that the association may have been driven by unmeasured individual characteristics such as hostility. That is, hostile individuals are less likely to perceive their workplace as trustworthy, and they are less likely to quit smoking. When individual responses are aggregated up to the work-unit level, this bias will tend to become "washed out". In our study, however, individual perceptions were not associated with smoking, while the multilevel results suggested the presence of a "contextual" influence of company-level trust on smoking, even after controlling for individual perceptions of trust.

Company-level trust can be hypothesized to influence employees' smoking in several ways. First, cohesive workplaces are likely to be more effective in maintaining healthy norms, e.g., encouraging workers to comply with medical advice following annual health check-ups and to adopt anti-smoking norms [1]. It is possible that workplaces with higher norms of trust can exert social control over deviant health-related behaviors, increasing concern for the potential adverse health effects and discomfort among coworkers exposed to second-hand smoke in the "close-knit" nature of these companies. Second, employees in more normative workplaces may find it easier to mobilize various forms of social support from coworkers. Third, cohesive workplaces may be more effective in sustaining collective action to reduce workplace health hazards, e.g., by implementing strict smoking restriction policies. When we additionally adjusted for the degree of implementation of anti-smoking management in companies, our coefficient estimates were largely unaffected, indicating that this was not a major explanation. Fourth, individual interactions with trusting coworkers may produce positive affective states, including a sense of security for being "accepted" within the company. Positive affective states are in turn hypothesized to increase motivation for self-care, such as smoking cessation [36,37].

There are two possible explanations for the lack of association between reciprocity and smoking both at individual- and company-levels. First, the referent area of the assessment (i.e., company) could have been too broad to capture the degree of reciprocity exchanges occurring within the workplace. In contrast to generalized trust, reciprocity implies a close two-way interaction, with the expectation that the favor would be returned when needed [27]. Indeed, it would be rather unrealistic to expect something in return from coworkers belonging to work-units outside one's own. In line with this, the overall proportion of workers reporting reciprocity was generally lower (< 25%) compared with the proportion of workers reporting generalized trust. Second, reciprocity may be a less meaningful component of workplace social capital among Japanese workers. In Japan, most employees are expected to work in close cooperation with supervisors and coworkers, with consensus sought at each step. In this cultural context, it is not clear what is meant when workers respond that there is high (or low) reciprocity among each other. Further work is needed to understand who within the company benefits (or pays the cost) of reciprocity [38].

In the present study, the MOR of the empty model was 1.54, indicating that if we randomly select a person in a company with a higher probability of smoking, his/her odds of smoking is (in median) 1.54 times higher than that of a person in a company with a lower probability of smoking. This relatively large variance at company-level could well reflect the sampling procedure; we used a representative sample of workers from entire Okayama prefecture, and it has been reported that smoking prevalence in Okayama can vary substantially according to municipalities [39]. Further, smoking has been indicated to be strongly associated with socioeconomic status among Japanese adults [40], which may also explain the large variance of smoking at company-level. Since the relatively large variance at the company-level could also suggest the possibility that the association between company-level trust and smoking might have been driven by unmeasured company characteristics, future studies are warranted to examine these alternative explanations.

Limitations of the current study include the lack of a validated, comprehensive assessment of workplace social capital in the Japanese context. Although the single item measure of trust is very broadly accepted as being valid [41], future studies of workplace social capital may benefit from approaches such as whole social network mapping to clarify which forms of workplace social capital (e.g. horizontal vs. vertical) is likely to affect workers' health [5,38,42-44]. Second, most smokers become smokers during adolescence, some during early adulthood and very few later than that [7]. Thus, most smokers have established their habit by the time they join the work-force, which suggests that the most sensitive health outcome to examine is smoking cessation. Indeed, smoking behavior is a dynamic process, indicating the necessity to track the smoking history over a period of time. Further study warrants investigating the link between workplace social capital and smoking cessation by longitudinal study among smokers. Thirdly, it is possible that employers recruit workers who are more cooperative, trusting, and do not smoke. To the extent that company characteristics reflect employer choices, there is an endogeneity problem, which will be difficult to overcome even with longitudinal data [45]. Fourth, there is a possibility of selection bias caused by non-responses. If the companies with lower social capital tended to refuse to participate in the study, this possible selection bias may have led to the underestimation of the current findings of a contextual effect. Further, the company-level socioeconomic status was not available, which might partly explain the association between company-level trust and smoking [46]. If company-level socioeconomic status was also associated with company-level reciprocity, however, we would have observed the association between company-level reciprocity and smoking, which contradicts with the present finding. In addition, a significant portion of the subjects did not answer the social capital items (trust; 37%, reciprocity; 47%). Last, we did not assess social capital outside the workplace setting. Workplace social capital may be affected by social capital outside companies, and vice versa. Indeed, a previous study in Japan has shown the importance of considering the social networks at work as well as outside companies on workers' health [47].

## Conclusions

In conclusion, we have found that company-level mistrust is associated with higher likelihood of smoking among Japanese private sector employees, while individual perceptions of mistrust did not appear to have an association. By contrast, no clear associations were found between lack of reciprocity and smoking both at the individual- and company-level. Further studies are warranted to examine the possible link between company-level trust and smoking cessation in the Japanese workplace.

## Competing interests

The authors declare that they have no competing interests.

## Authors' contributions

ES conceived of the study, and participated in its design and performed the statistical analysis and drafted the manuscript as a principal author. TF helped in interpreting the results and writing the manuscript. ST conceived of the study, and participated in its design and critically revised the manuscript for intellectual content. SVS and EY helped in the data analysis, contributed to interpretation of the results, and critically revised the manuscript for intellectual content. IK helped in interpreting the results and edited the manuscript. All authors read and approved the final manuscript.

## Pre-publication history

The pre-publication history for this paper can be accessed here:

http://www.biomedcentral.com/1471-2458/10/489/prepub
